# Coal gangue recognition based on spectral imaging combined with XGBoost

**DOI:** 10.1371/journal.pone.0279955

**Published:** 2023-01-19

**Authors:** Minghao Zhou, Wenhao Lai

**Affiliations:** 1 Inner Mongolia University of Technology, School of science, Hohhot, China; 2 Anhui University of Science and Technology, School of Electrical and Information Engineering, Huainan, China; Wroclaw University of Science and Technology, POLAND

## Abstract

The identification of coal gangue is of great significance for its intelligent separation. To overcome the interference of visible light, we propose coal gangue recognition based on multispectral imaging and Extreme Gradient Boosting (XGBoost). The data acquisition system is built in the laboratory, and 280 groups of spectral data of coal and coal gangue are collected respectively through the imager. The spectral intensities of all channels of each group of spectral data are averaged, and then the dimensionality is reduced by principal component analysis. XGBoost is used to identify coal and coal gangue based on the reduced dimension spectral data. The results show that PCA combined with XGBoost has the relatively best classification performance, and its recognition accuracy of coal and coal gangue is 98.33%. In this paper, the ensemble-learning algorithm XGBoost is combined with spectral imaging technology to realize the rapid and accurate identification of coal and coal gangue, which is of great significance to the intelligent separation of coal gangue and the intelligent construction of coal mines.

## 1. Introduction

The proportion of coal in China’s primary energy will gradually decrease in the process of achieving the goal of ‘Carbon Peak and Carbon Neutrality’ [1, 2]. In the process of coal mining, coal gangue will be mixed, which affects the combustion efficiency of coal. Gangue [3], which is a companion of coal mining, is a kind of stone with low combustion value or even non-combustibility. Therefore, it is necessary to separate coal gangue from raw coal [4, 5]. China’s coal industry has entered a new stage of intellectualization [6], which makes the intelligent separation of coal gangue more and more meaningful.

Rapid and efficient separation of coal gangue can improve the quality of raw coal [7]. The recognition of coal gangue in raw coal mainly includes the ray and imaging methods. Kong et al. [8] proposed coal gangue identification based on dual-energy γ rays in 1997. The equipment of this method is complex and expensive, and it can endanger the health of workers. With the popularity of computers and high-performance imaging equipment, coal gangue recognition based on imaging has become the mainstream research direction of coal gangue intelligent separation. He Min et al. [9] studied the recognition of coal and coal gangue by using six artificial features such as gray mean in 2012. Shen et al. [10] built a gangue image acquisition device in 2019 to identify gangue with more complex artificial features but still could not avoid the problem of robustness and insufficient expression of artificial features. Zhao et al. [11]established a PSO-SVM model for the classification of coal and gangue in 2022. Deep learning has improved pattern recognition technology in the field of speech and vision. In addition, a convolutional neural network (CNN) has made a breakthrough in image recognition [12, 13]. It provides a new idea for the identification of coal gangue [14–17]. In 2020, Xu et al. [18] studied gangue recognition in an end-to-end manner based on deep convolutional neural networks. In 2021, Xing et al. [19] proposed a coal gangue identification method based on lidar and DenseNet intensity images. The recognition of Coal Gangue Based on deep learning overcomes the shortcomings of artificial features, but it has strict requirements for computational performance.

At present, the recognition of coal gangue based on imaging is mainly RGB, which is easy to be interference by visible light. Spectroscopy is the study of matter or energy using the phenomenon of light, sound, or particles emitted, absorbed, or reflected by substance. It is defined as the study of the interaction between electromagnetic waves of different wavelengths and matter. Multispectral imaging (MSI) [20, 21] is an imaging system of spatial imaging combined with spectral detection. It uses imaging technology to obtain image data of multiple narrow-band spectra. Compared with RGB imaging, multispectral imaging collects images of several different band regions, which have both spectral information and spatial information [22]. It effectively avoids the problems of narrow bands and easy interference of traditional RGB images. The imaging time of multiple spectra is short compared to hyperspectral. So, its real-time performance is better [23]. With the development of technology, multispectral technology is widely used in biomedicine [24, 25], agricultural production [26, 27], food detection [28, 29], color identification [30, 31], archaeology [32–34], ecological protection [35, 36], etc. Hu et al. [37] proposed a new solution to classify coal and coal gangue from multispectral images. He used a features extraction algorithm and SVM to build the recognition model, which verified the feasibility of using multispectral to identify coal gangue, but it only used the single band of multispectral. Lai et al. [38] proposed to use multispectral imaging technology for the intelligent separation of gangue, select the best-combined bands by OIF, and design a detection model to achieve rapid identification and localization of gangue. He further verified the advanced technology of using multispectral to identify coal gangue. Their research uses only part of the multispectral data. The number of multispectral bands is smaller than that of hyperspectral, but it still has more than ten or even dozens of bands. The fast and efficient identification of coal gangue based on multispectral imaging needs to be further studied.

Extreme Gradient Boosting (XGBoost) is a newer ensemble-learning algorithm proposed by Chen et al. [39] in 2016. It is highly learnable, friendly, and interpretable to high-dimension data, and has shown high usability and performance in many data science competitions. The reliability of XGBoost training is good and the computational efficiency of its model will not decline seriously with the expansion of the training sample size [40]. It has been widely used in data analysis in medical care [41], finance [42, 43], etc. Based on the excellent high-dimensional data processing and learning ability of XGBoost, it is combined with multi-spectral imaging technology to identify coal gangue.

Principal Component Analysis (PCA) is a widely used data dimension reduction algorithm that reduces the number of sample features and improves the computational efficiency of classification models [44]. We propose coal gangue recognition based on XGBoost and multispectral imaging in this paper. To reduce the interference of redundant data as much as possible and improve the identification efficiency of coal gangue. We also use PCA to reduce the dimension of multispectral data, to realize the rapid and accurate identification of coal and coal gangue. The research in this paper provides a new solution for the rapid and accurate identification of coal gangue, enriches the application field of multispectral imaging, and is of great significance for the clean and efficient utilization of coal resources and the intelligent construction of coal mines.

## 2. Materials and methods

### 2.1 Equipment and materials

From a spatial perspective, traditional spectroscopic techniques are mostly for the detection of a single-point location, that is, a single-point spectrometer, while spectral imaging is a combination of spectroscopic technology and imaging technology. Spectral imaging technology makes full use of the absorption or radiation characteristics of substances to different electromagnetic spectra and adds one-dimensional spectral information on the basis of ordinary two-dimensional spatial imaging. The imaging spectrum can obtain image information and spectral information of pixels, which can be divided into multispectral and hyperspectral according to spectral resolution.

**Fig 1:**
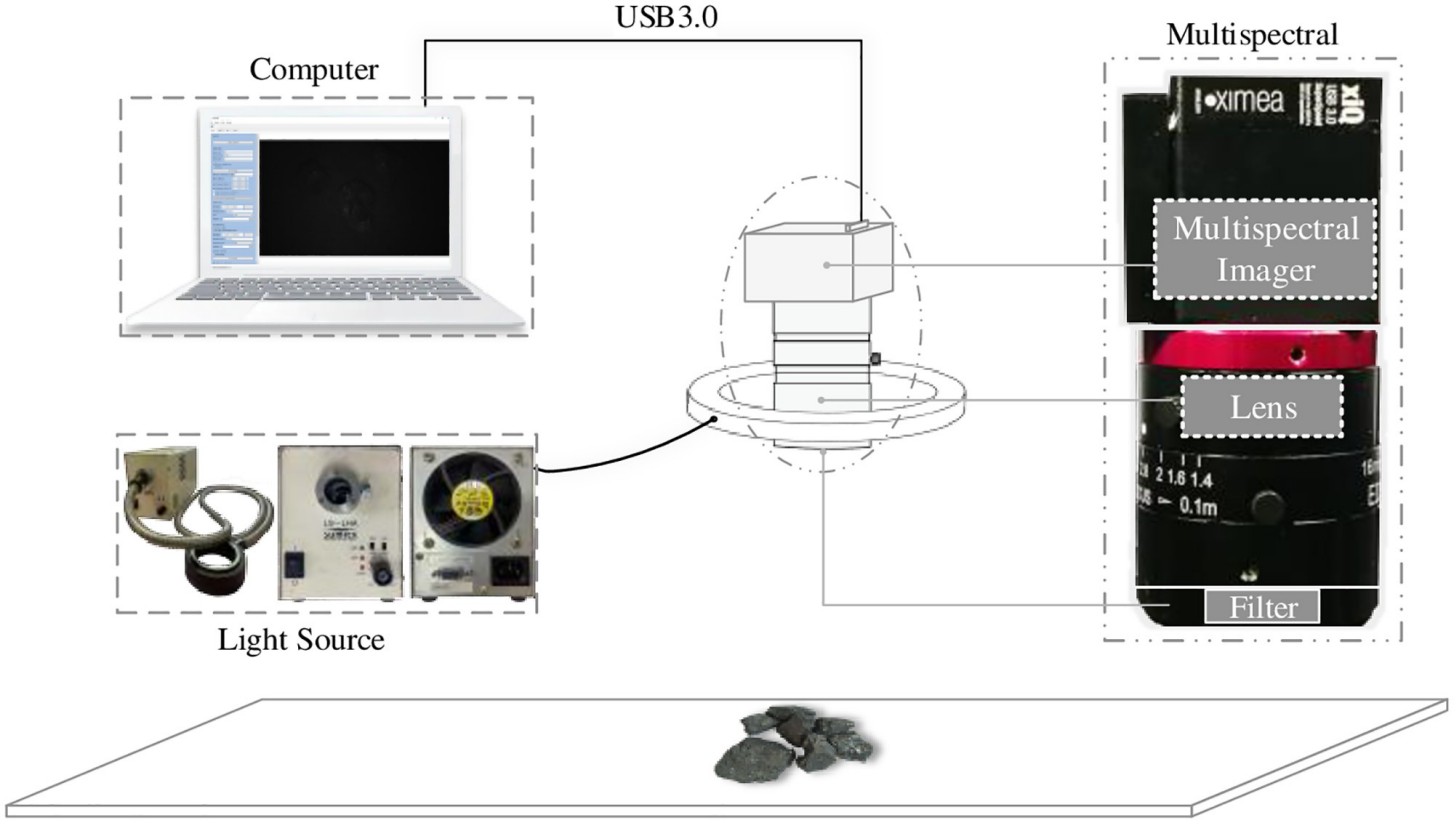
Core equipment of data acquisition system.

Multispectral imaging has both spectral and image information, and the imaging time is relatively fast. Therefore, multispectral imaging is used for coal gangue recognition in this paper. The core components of the spectral imaging system in this paper include lens, multispectral imager, filter, and light source. Build a multispectral data acquisition system of coal and coal gangue in the laboratory. The selected equipment is shown in Fig 1.

The device in Fig 1 is a lens, an imager, a filter, and a light source from left to right. The spectral imager is produced by the German company XIMEA and has 25 bands. The filter is a product of Edmund optics, which allows light with near-infrared wavelength to pass through. The light source is LS-LHA produced by a Japanese company.

**Fig 2:**
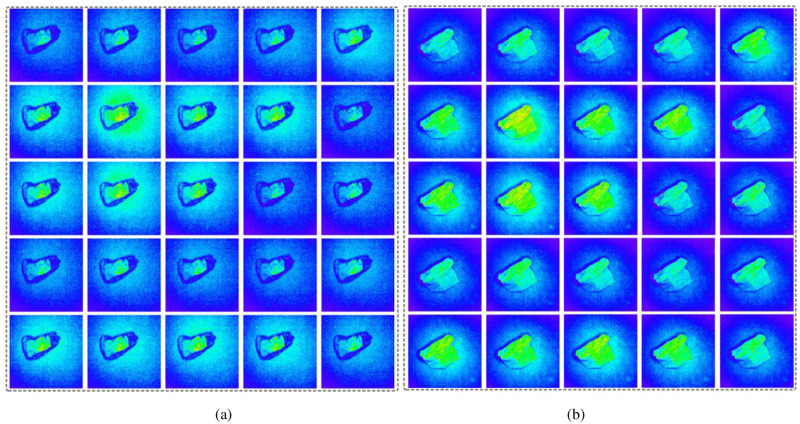
Multispectral images of coal and coal gangue. (a) Spectral image of coal; (b) Spectral image of coal gangue.

The multispectral imager used in the experiment has a total of 25 channels. Each spectral channel corresponds to one spectral image, which means that a group of multispectral data contains 25 spectral images. The resolution of the raw spectral image is 409×216 pixels. Part of the resolution of each group of spectral data is randomly selected for coal gangue identification to reduce the amount of calculated data. The image resolution of selected spectral data for each channel is 200×200 pixels. The multispectral images of coal and coal gangue are shown in Fig 2.

Fig 2(a) shows 25 spectral images of a group of multispectral data of coal. Fig 2(b) shows 25 spectral images of a group of multispectral data of coal gangue. It can be seen from the comparison in the figure that there are differences in the spectra of coal and coal gangue, and the spectral intensity of the same coal or gangue in different channels also has differences.

In this experiment, 560 groups of multispectral data of coal and coal gangue were collected, of which 440 groups were used as training set for training models and others for testing models.

### 2.2 Method

#### 2.2.1 Principal component analysis

Principal Component Analysis (PCA) is used to reduce the dimension of multispectral data, and then it is used for XGBoost to identify coal gangue. PCA is one of the most important and widely used dimension reduction algorithms. Its core idea is to project the data into a new space so that the N-dimension features are mapped to the K-dimension(K<N). The steps of PCA dimension reduction data are as follows:

Step 1: Normalize the data;
Step 2: Calculate the covariance matrix of all sample features;
Step 3: Calculate the eigenvalues of the covariance matrix;
Step 4: Calculate the eigenvectors corresponding to the eigenvalues;
Step 5: Select the eigenvectors corresponding to the largest K eigenvalues to form a new matrix;
Step 6: The data is time the eigenvector matrix to achieve dimensionality reduction of the data.

#### 2.2.2 XGBoost theory

Chen et al. proposed the XGBoost algorithm. It is essentially a gradient-boosting decision tree. Compared with other ensemble learning algorithms, XGBoost adds a regularization mechanism to prevent the overfitting of the model. XGBoost uses both first-order and second-order derivatives and supports user-defined loss functions; When finding the best segmentation point, an approximation method is realized, and the processing of sparse data sets and missing values are also considered, which greatly improves the efficiency of the algorithm. XGBoost is a forward feature algorithm, and its mathematical model as:

$${\hat{y}}_{i}=\sum_{k=1}^{K}f_{k}(x_{i})\;,\;\;\;f_{k}\in F$$ (1)

where, $\hat{y}$ is the prediction result, *K* is the number of trees, *f* is a regression tree model, *f*(*x*) = *ω*_*q*(*x*)_, *q*:*R*^*m*^ → *T*, *ω* ∈ *R*^*T*^, *T* is the number of leaves in the tree, and *F* is the function space composed of all regression trees. *q* represents the structure of each tree, it maps a training sample instance to the corresponding leaf index.

$$obj^{t}=\sum_{i=1}^{n}l\left(y_{i},{\hat{y}}_{i}\right)\;+\sum_{i=1}^{t}\Omega \left(f_{i}\right)$$ (2)

$$\Omega \left(f\right)=\gamma T+\frac{1}{2}\lambda {\left\|w\right\|}^{2}$$ (3)

where, *l*(·) is the loss function and it is a differentiable convex function. Ω(*f*_*i*_) is a regularization item that suppresses the complexity of the model. $\sum_{i}^{t}\Omega \left(f_{i}\right)$ is the sum of the complexity of all *t* trees, it is used to prevent overfitting of the XGBoost. *γ* is a parameter that controls the number of leaf nodes and *λ* controls that the score of leaf nodes.

XGBoost is essentially a boosting algorithm. The predicted result of the *i*-th sample *x*_*i*_ by the model in the *t*-th step can also be recorded as:

$${\hat{y}}_{i}^{t}={\hat{y}}_{i}^{t-1}+f_{t}\left(x_{i}\right)$$ (4)

where, ${\hat{y}}_{i}^{t-1}$ is the prediction result of the model output in step *t*-1. Therefore, the objective function of the model can be recorded as:

$$obj^{t}=\sum_{i=1}^{n}l\left(y_{i},{\hat{y}}_{i}^{t-1}+f_{t}(x_{i})\right)\;+\sum_{i=1}^{t}\Omega \left(f_{i}\right)$$ (5)

The goal is to find the tree that minimizes the loss of model output on training data in the *t* step. This tree is the best tree we are looking for in this step. The second-order approximation can be used to quickly optimize the objective function. After that, we can get the objective function for further optimization.

$$obj^{t}≃\sum_{j=1}^{T}\left[\left(\sum_{i\in I_{j}}g_{i}\right)w_{j}+\frac{1}{2}\left(\sum_{i\in I_{j}}h_{i}+\lambda \right)w_{j}^{2}\right]+\gamma T$$ (6)

where, *w*_*j*_ is the *j*-th leaf node value. All *x* of the *j*-th leaf node are split into a set of leaf nodes recorded as *I*_*j*_ = {*i*|*q*(*x*_*i*_) = *j*}. $g_{i}=\partial_{\hat{y}\left(t-1\right)}|l\left(y_{i},{\hat{y}}_{i}^{t-1}\right)$ and $h_{i}=\partial_{\hat{y}\left(t-1\right)}^{2}|l\left(y_{i},{\hat{y}}_{i}^{t-1}\right)$ are the first derivative and the second derivative, respectively.

Let:

$$H_{j}=\sum_{i\in I_{j}}h_{i}$$ (7)

$$G_{j}=\sum_{i\in I_{j}}g_{i}$$ (8)

where, *G*_*j*_ is the sum of the first-order partial derivatives of the samples included in leaf node *j*. *G*_*j*_ and *H*_*j*_ are the results obtained in the previous *t*-1 step, and their values are known and can be regarded as constant. Therefore, only the leaf node *w*_*j*_ of the last tree is a variable in Eq 6. Assuming that the optimal weight of the *j*-th leaf is $w_{j}^{*}$, it is calculated as shown in Eq 9.


$$\sum_{i\in I_{j}}g_{i}+\left(\sum_{i\in I_{j}}h_{i}+\lambda \right)w_{j}^{*}=0$$ (9)

In the process of model training, when building the *t*-th tree, a core problem is how to find the optimal split of a leaf node. XGBoost has two methods for splitting nodes, namely the exact greedy algorithm and the approximation algorithm. In this paper, we choose exact greedy algorithm to split the tree.

Assuming that we complete feature splitting at a node, the objective function before splitting is recorded as:

$$obj_{1}=-\frac{1}{2}\left[\frac{{\left(G_{L}+G_{R}\right)}^{2}}{H_{L}+H_{R}+\lambda }\right]+\gamma$$ (10)

The objective function after splitting is recorded as:

$$obj_{2}=-\frac{1}{2}\left[\frac{G_{L}^{2}}{H_{L}+\lambda }+\frac{G_{R}^{2}}{H_{R}+\lambda }\right]+2\gamma$$ (11)

## 3. The study of coal gangue recognition

### 3.1 Dimensionality reduction of spectral data

Although the resolution of the spectral image of the obtained multispectral data is just 200×200 pixels, each group of spectral data includes 25 channels. Each group of multispectral data is used for PCA dimension reduction without preprocessing, which will cause PCA to consume a lot of computing resources. The spectral data of 25 channels in each group of coal and coal gangue spectral data were averaged.

**Fig 3:**
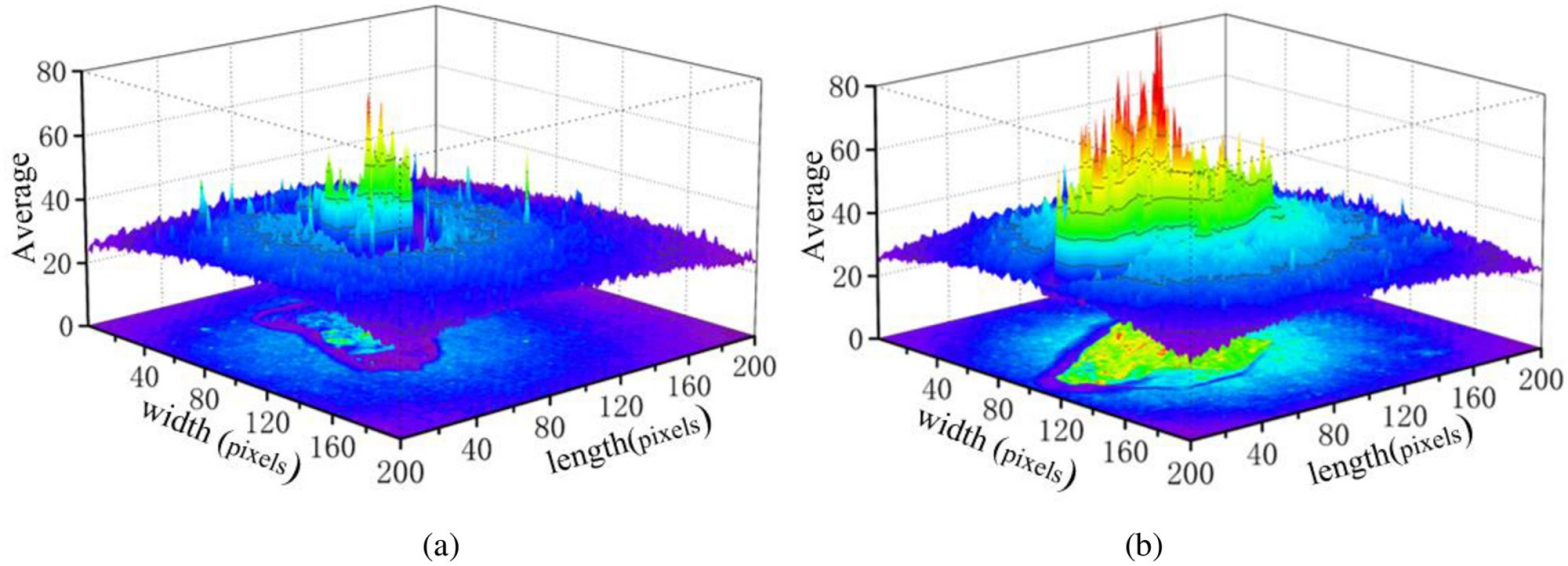
Average of 25 bands of spectral image data. (a) Coal; (b) Coal gangue.

Fig 3 shows the average of a group of coal or coal gangue spectral data. The resolution of the average spectral intensity image is also 200×200 pixels, which is converted into one-dimensional vector data, and then the PCA algorithm is used for dimensionality reduction. There are 280 groups of spectral data for coal and coal gangue, respectively. The division of training set and test set is shown in Table 1.

**Table 1:** Division of experimental data.

Classes	Number of training set samples	Number of test set samples	*total*
Coal	220	60	280
Coal gangue	220	60	280
*total*	440	120	560

**Fig 4:**
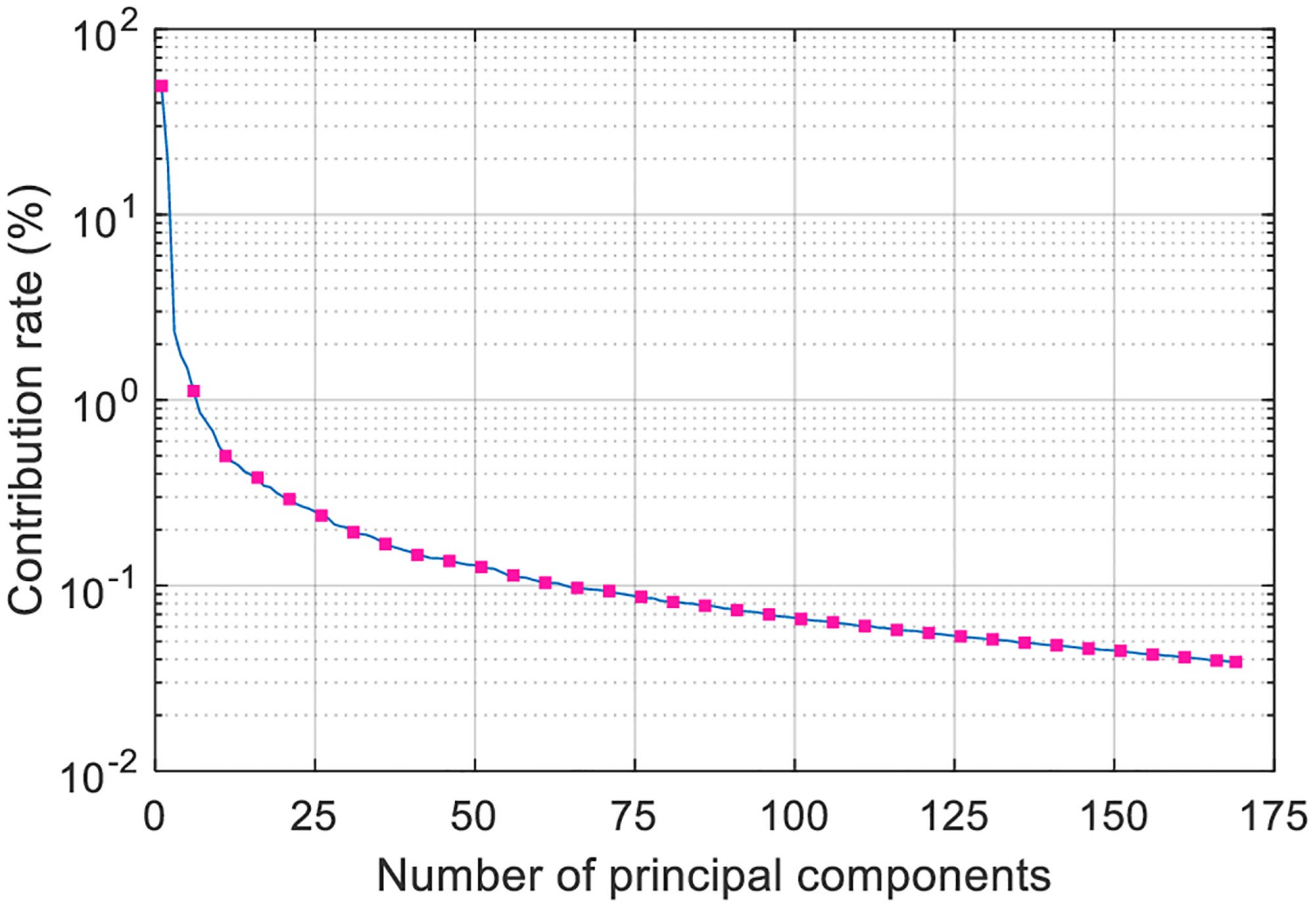
Principal component contribution rate.

We use PCA for training data dimension reduction. The number of principal components corresponding to the cumulative contribution rate higher than 95% is 169, and the contribution rate of each principal component is shown in Fig 4.

The maximum contribution rate of a single principal component of the multispectral data after PCA dimension reduction in Fig 4 is about 50%. The contribution rate of the seventh principal component is less than 1%, and the number of principal components whose cumulative contribution rate is higher than 75%, 80%, 85%, and 90% is 7, 16, 36, and 80, respectively.

### 3.2 Recognition of coal gangue based on XGBoost

The performance of XGBoost is excellent, but its parameters must be set to appropriate values. Compared with other machine learning algorithms such as decision trees or random forests, XGBoost has relatively more parameters to be optimized. Optimizing all parameters will increase consumption. Our strategy is to set the values of some parameters based on experience. The number of weak learning machines is 100, the sample weight of the minimum leaf node is 2, the maximum depth of the tree is 3, and the learning rate is 0.25. Other parameters that need to be set appropriately are optimized through an algorithm. So, the parameters gamma, column sampling rate, and down-sampling rate settings are optimized by a meta-heuristic algorithm.

**Fig 5:**
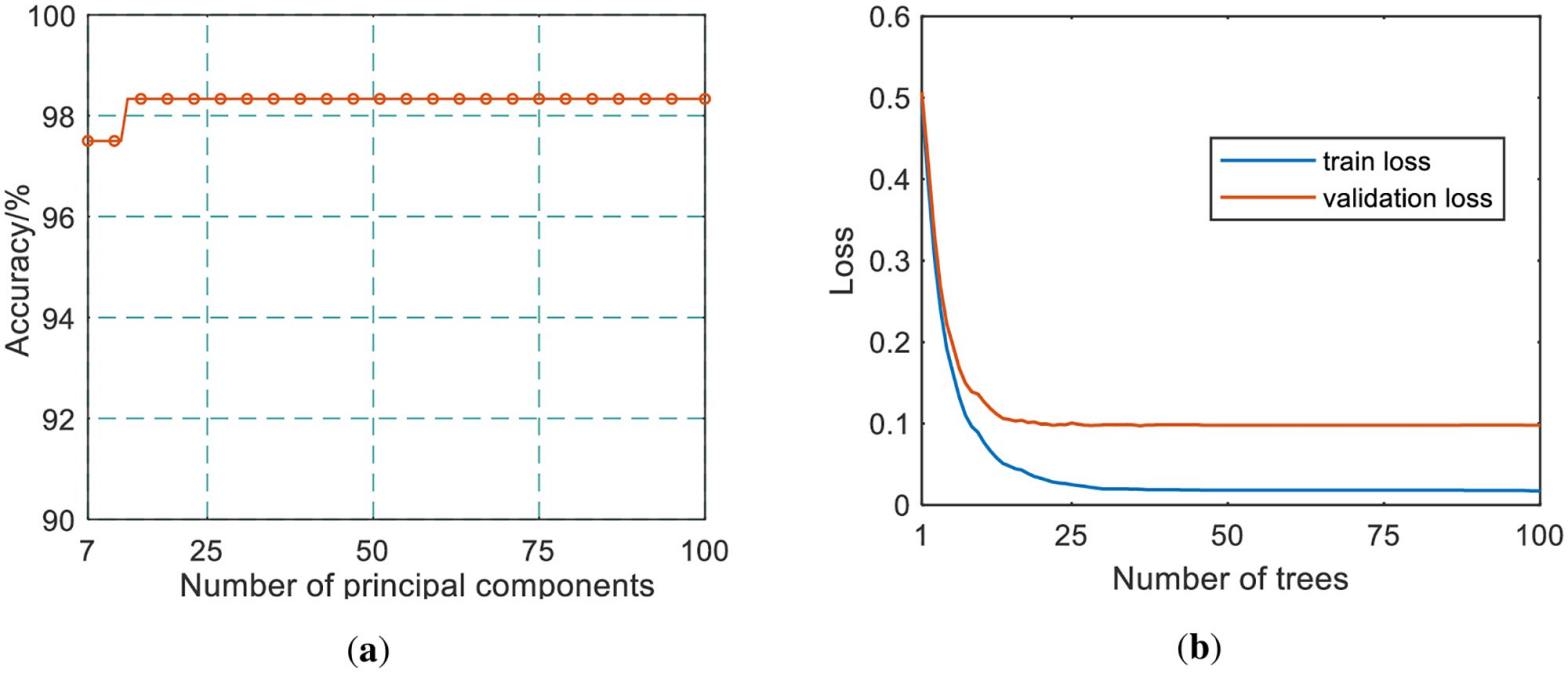
Coal and coal gangue identification results of XGBoost. (a) Accuracy; (b) Loss.

We use the dimension-reduced spectral data and XGBoost algorithm for coal gangue identification research. And the number of principal components also needs to be set reasonably. When the cumulative contribution rate of principal components is higher than 75%, and the number of principal components takes different values, the recognition accuracy of XGBoost for coal and coal gangue recognition is shown in Fig 5.

It can be seen from Fig 5 that when the number of principal components is 13, the recognition accuracy of XGBoost for coal and coal gangue does not increase with the number of principal components. Considering the contribution rate of principal components, the recognition accuracy of coal and coal gangue, and XGBoost efficiency, the number of principal components of dimension reduction spectral data is set to 16, which cumulative contribution rate is higher than 80%.

Fig 5(b) shows the training loss and validation loss of XGBoost when the number of principal components is 16. It can be seen from the loss curve that XGBoost has a better learning ability to identify coal and coal gangue based on dimension-reduced spectral data. After about 20 weak learning machines were studied, the training and verification losses have been reduced to less than 0.1. Although the validation loss is greater than the training loss, as the training loss decreases, the validation loss does not tend to increase, which means that XGBoost has not been overfitted during training.

The Classification and Regression Tree (CART), Adaptive Boosting (AdaBoost), K-Nearest Neighbor (KNN), and Extreme Learning Machine (ELM) are also used for coal and coal gangue identification to verify the coal and coal gangue recognition performance of XGBoost. The number of AdaBoost weak learning machines, ELM hidden layer nodes, and KNN neighbors are 100, 100, and 5, respectively.

**Fig 6:**
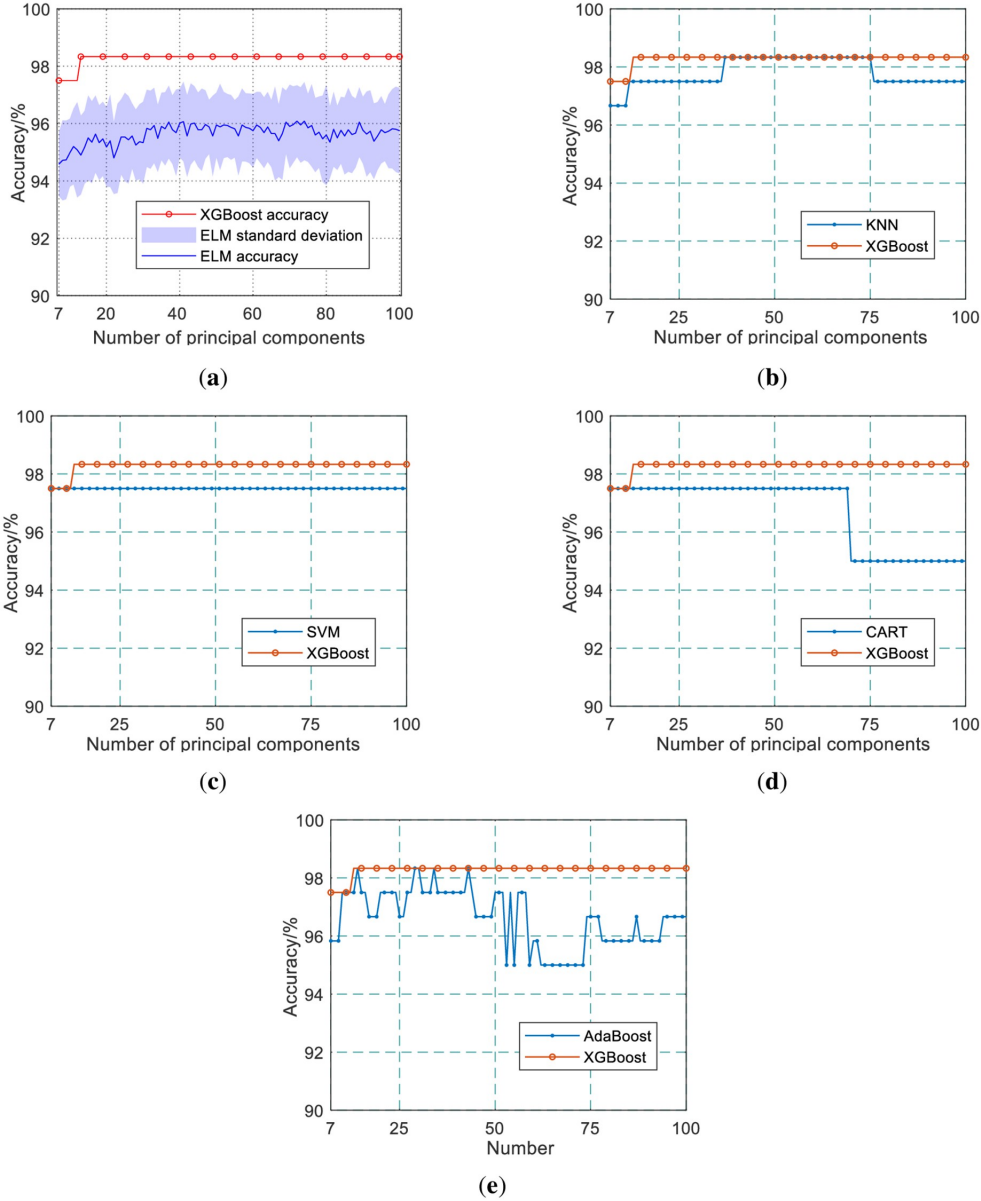
Recognition results of different algorithms. (a) ELM; (b) KNN; (c) SVM; (d) CART; (e) AdaBoost.

The initial weights of the ELM are random, which leads to the possibility that the results of each independent experiment may not be the same. Therefore, we plot its standard deviation in Fig 6a.

(a), (b), (c), (d), and (e) in Fig 6 are the comparisons of the recognition results of ELM, KNN, SVM, CART, and AdaBoost with XGBoost, respectively when the principal components of the spectral data take different values after dimensionality reduction by PCA. Since some of the initial weights of ELM are random, the training results on the same data may be different. Therefore, when the principal components take different values, the experiment is repeated 50 times, and the results are averaged.

**Fig 7:**
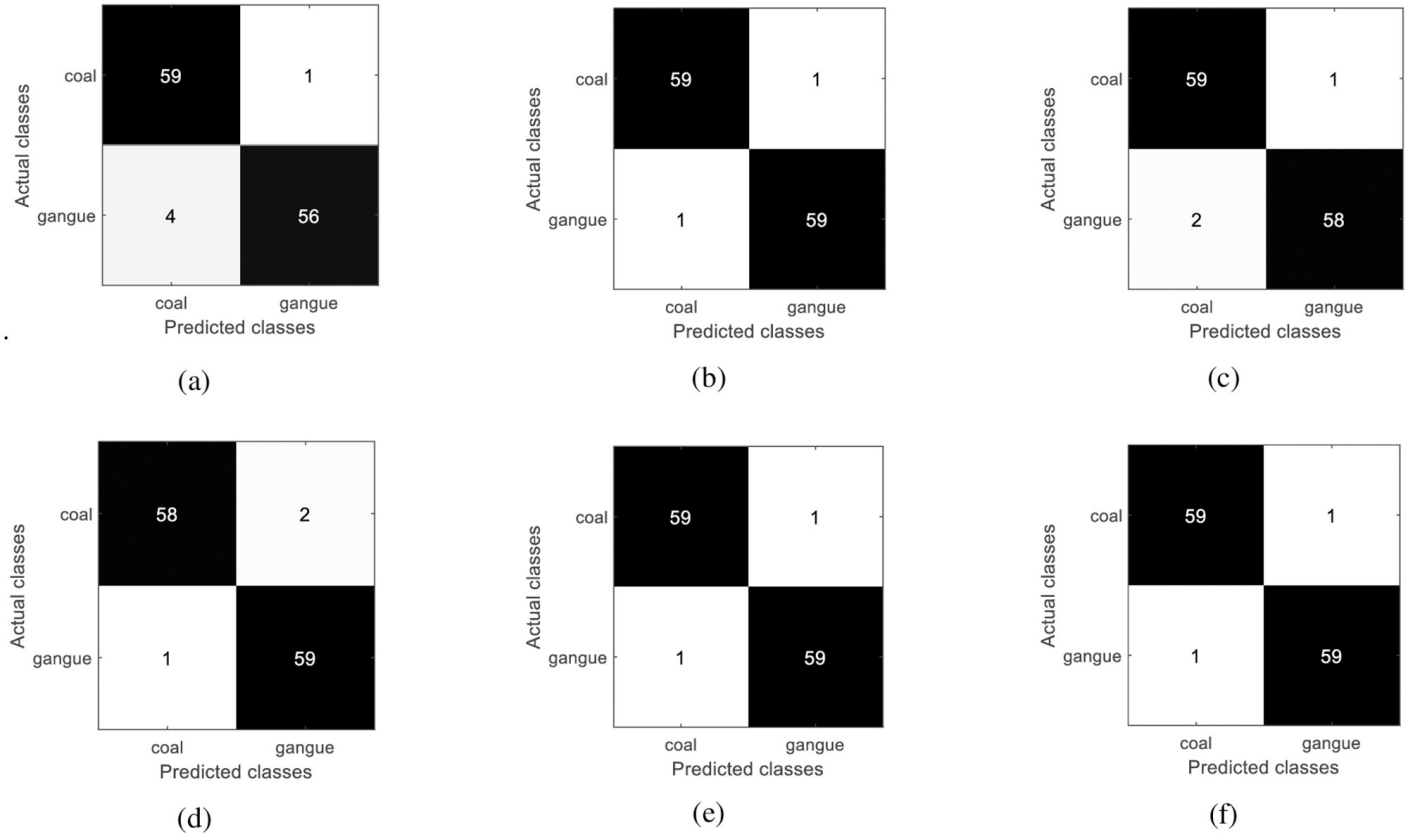
Test confusion matrix of different algorithms. (a) ELM; (b) KNN; (c) SVM; (d) CART; (e) AdaBoost; (f) XGBoost.

It can be seen that when the cumulative contribution rate of principal components is higher than 80%, the recognition accuracy of SVM for coal and coal gangue does not change with the increase of the number of principal components. And the accuracy of AdaBoost in identifying coal and coal gangue is more sensitive to the number of principal components. The test confusion matrix of the best recognition results of different algorithms is shown in Fig 7.

F1 score is an indicator used to measure the accuracy of the model in statistics, which takes into account both the precision and recall of the classification model. The realization mechanism of F1 score is as follows:

$$F_{1}=2\times \frac{p_{r}\times r_{e}}{p_{r}+r_{e}}$$ (12)

where, *p*_r_ and *r*_e_ are precision and recall respectively. We can obtain the F1 scores and test accuracy according to the confusion matrix. The optimal recognition results of different algorithms are shown in Table 2. “Accuracy”is the number of accurately predicted samples divided by the total number of samples, which is the macro accuracy.

**Table 2:** The comparison of training and testing results of different algorithms.

Algorithm	Data dimension reduction				Mean spectral data		
	Train accuracy (%)	Test accuracy (%)	F1	Principal components	Train accuracy (%)	Test accuracy (%)	F1
XGBoost	100.00	98.33	0.983	16	100.00	98.33	0.983
CART	99.32	97.50	0.975	16	98.86	84.17	0.846
AdaBoost	100.00	98.33	0.983	29	100.00	96.67	0.967
ELM	99.09	95.83	0.959	16	92.73	85.83	0.858
KNN	99.32	98.33	0.983	38	98.41	95.83	0.959
SVM	99.09	97.50	0.975	16	71.59	65.00	0.705

In Table 2, “Principal components” is the number of principal components. When the cumulative contribution rate of principal components is greater than 80%, the minimum number of principal components corresponding to the maximum accuracy of CART, AdaBoost, ELM, KNN, and SVM recognition coal and coal gangue are 16, 29, 16, 38, and 16, respectively. The recognition accuracy of coal and coal gangue of XGBoost, AdaBoost, and KNN is 98.33%. However, the number of principal components of XGBoost is less than that of the other two algorithms, and the number of principal components is only 16.

“Mean spectral data” is the average spectral intensity data of 25 bands without dimensionality reduction, and the data dimension is 1×40000. For the average spectral data without dimensionality reduction, XGBoost has the highest recognition accuracy for coal and coal gangue, which is 98.33%. The classification ability of SVM for high-dimensional data is relatively weak, and the classification performance is more sensitive to the setting of parameters. Therefore, the recognition accuracy of SVM for dimension unreduced spectral data is the smallest, which is 65.00%.

In the Table 2, the recognition accuracy of CART, AdaBoost, ELM, and KNN has been significantly improved, which means that the spectral data can effectively reduce redundant information after PCA dimensionality reduction. It can also be known that XGBoost has the highest accuracy for coal and coal gangue recognition, regardless of whether the dimension of spectral data is reduced or not.

K-Fold cross validation is a way to verify model performance in machine learning. In this paper, we randomly divide the collected experimental data, instead of using K-Fold cross validation, and XGBoost shows relatively good performance in the given training and test set. The verification of model performance is not comprehensive, but it shows the advantages of XGBoost to a certain extent.

### 3.3 Comparison and analysis

The data before dimensionality reduction is the average spectral intensity of 25-channel spectral images, and its essence is also an image with a resolution of 200×200 pixels. Based on the average spectral data, this paper uses artificial features combined with an ensemble learning algorithm to identify coal and coal gangue, and the results are used for comparison. The artificial feature of the spectral images in this paper is Local Binary Pattern (LBP), and the ensemble learning algorithm uses AdaBoost algorithm to identify coal and coal gangue. The recognition results of different spectral data processing methods are shown in Table 3.

**Table 3:** Recognition results of different data processing methods.

	XGBoost					AdaBoost				
	Train accuracy (%)	Train time (s)	Standard deviation (s)	Test accuracy (%)	Test F1score	Train accuracy (%)	Train time (s)	Standard deviation (s)	Test accuracy (%)	Test F1score
PCA	100.00	0.05533	0.00337	98.33	0.983	100.00	0.12123	0.00152	97.50	0.975
LBP	100.00	0.07101	0.01379	90.83	0.913	96.81	0.23573	0.04202	83.33	0.840
None	100.00	7.68853	0.2053	98.33	0.983	100.00	42.7931	0.40627	95.00	0.950

In Table 3, ’PCA’ refers to the dimensionality reduction of the average spectral data by principal component analysis, and the number of principal components is 16. "Train time" is the average training time of 50 independent experiments, and “Standard deviation” is its corresponding standard deviation. “F1score” is the F1 score of each algorithm on the test data set. LBP represent the average spectral data processed with artificial features, and “None” is the average spectral data without processing.

Compared with the artificial feature LBP, AdaBoost and XGBoost have the relatively highest recognition accuracy for coal and coal gangue after the spectral data is dimension reduced by PCA. Although XGBoost uses dimension-reduced and non-dimension-reduced data to identify coal and coal gangue with the same accuracy. However, after PCA dimension reduction, the training time used by XGBoost is less than that used without PCA processing, which is 0.05533 seconds. This means that using PCA to reduce spectral data can greatly improve the efficiency of XGBoost.

Using the same data processing method, XGBoost has the best recognition accuracy for coal and coal gangue, and the efficiency of XGBoost is higher than that of AdaBoost. By comparison, it can be seen that XGBoost combined with PCA has relatively better performance in identifying coal and coal gangue based on multispectral data.

## 4. Conclusions

The rapid and accurate identification of coal and coal gangue is of great significance to the clean and efficient utilization of coal and the intelligent construction of coal mines. In this paper, we propose multispectral imaging combined with the XGBoost algorithm for coal gangue identification to overcome the shortcomings of RGB images that are easily interfered with by ambient light. The spectral intensity of 25 channels is averaged, and PCA is used to reduce the dimension of spectral data. Besides, combining PCA with ELM, KNN, SVM, CART, and AdaBoost is also used for coal gangue identification. The results show that PCA combined with XGBoost can achieve high-accuracy identification of coal gangue while consuming relatively less time.

## Supporting information

S1 Data (XLSX)
